# Developing an Educational Package to Improve Attitude of Medical Students Toward People With Mental Illness: A Delphi Expert Panel, Based on a Scoping Review

**DOI:** 10.3389/fpsyt.2022.860117

**Published:** 2022-03-14

**Authors:** Farahnaz Rezvanifar, Seyed Vahid Shariat, Mohammadreza Shalbafan, Razieh Salehian, Maryam Rasoulian

**Affiliations:** Mental Health Research Center, Psychosocial Health Research Institute, Department of Psychiatry, School of Medicine, Iran University of Medical Sciences, Tehran, Iran

**Keywords:** social stigma, community psychiatry, mental illness, medical students, medical education

## Abstract

**Introduction:**

The importance of stigma toward patients with mental illness in medical students as future physicians cannot be overemphasized. There is currently no formal training to reduce stigma toward mental illness in medical students in their educational curriculums in Iran like most other low and middle income countries. Therefore, aiming to provide a practical and effective training package focused on reducing stigma toward patients with mental illness in medical students, the current study conducted, as an expert panel with Delphi method, based on a scoping review, to develop an education package to improve attitude of medical students toward patients with mental illness.

**Materials and Methods:**

We surveyed the available international databases including PubMed, Google Scholar, Scopus, PsycINFO, Tripdatabase, Web of Science, Cochrane Database of Systematic Reviews as well as Persian databases including Iranmedex, SID, Irandoc and Magiran in February and March 2020. After an extensive review of related resources, 13 articles met our inclusion criteria. Then, we extracted the related data including type and duration of the interventions, sample size, mean and standard deviation of stigma scores before and after interventions. To develop the package among the included interventions, we asked 16 experts in psychology, psychiatry, and social medicine to rate the interventions based on a number of variables such as effectiveness, feasibility and applicability in a Delphi process.

**Results:**

The selected intervention in Delphi method with consensus of experts included a set of four sequential interactive interventions: showing a movie and discussing it, psychiatric training including contact with people who affected psychiatric disorders, social communication with people who affected psychiatric disorders, and group discussion on defining stigma and personal experiences.

**Conclusion:**

In the present study, we recommend a set of interventions to reduce stigma toward patients with mental illness among medical students in the form of a package of combined, interactive and sequential interventions that have been previously been shown to be effective in reducing stigma related to mental illness. We expect that implementation of these interventions would reduce mental illness stigma in medical students; which needs further verification.

## Introduction

Stigma is a negative form of labeling individuals or a group of individuals that distinguishes them from the other members of the society, based on physical or psychological differences or perceived differences (1–3). The word stigma has Greek roots, referring to a marking that was seared on the foreheads of the slaves in the past to distinguish them from others and prevent them from escaping (1, 4). In Sanskrit language, too, the Aryan word, stigma, means to mark (5). The stigma of mental illness leads to discrimination, loss of social status, confrontational behaviors, and reduced quality of life for those labeled mentally ill. For the stigmatized individual, this can aggravate the illness, result in substance abuse, prevent them from taking medications and not following up with treatment, and ultimately create serious issues for their families (6). On the other hand, from the public's point of view, admitted people with psychiatric disorders are perceived be different from other hospitalized people. This different position causes emotional distress in people with psychiatric disorders. This difference of attitude toward psychiatric disorder is because a patient hospitalized in the psychiatric ward, especially a person with schizophrenia, is perceived by others as dangerous, incompetent and unreliable (7–9).

Stigmatized attitude toward patients with mental illness is also a major problematic concern among healthcare workers including medical students. One of the main negative attitudes toward mental illness by physicians with different specialties is that they consider the psychotropic medications as dangerous, ineffective, and with many side effects that endanger their patients' independence, while physicians play a key role in introducing psychiatry to people and changing their attitudes (10).

In addition to the prominent and well-established role of mental health therapists in treating mental disorders, their actions are also crucial in reducing stigma. Among the actions of psychologists and psychiatrists in de-stigmatizing psychiatric people is improving the quality of psychiatric services. Providing comprehensive and team therapies is one of these strategies. For example, holding classes and workshops for the public and other health care providers or their presence in the media can reduce stigma (11). Timely provision of good mental health care services and effective treatment of mental illnesses can prevent the deterioration of patients and result in their better integration in society. This could have an important role in de-stigmatization of mental health conditions. In this regard, certain groups, including physicians, need to be trained (11). Unfortunately, lack of educational programs for physicians on psychiatric disorders and the negative attitude toward mental health conditions is prevalent among physicians and paramedics, which results in stigmatization of people with mental illness (12). Also, there are limited studies in Iran which have examined the stigma of mental illness among medical students. Tavakoli et al.'s (13) study on medical students demonstrated that cognitive and emotional components impact the formation of mental illness stigma, negatively impacting students' assessment of people with mental health conditions as risky and uncontrollable (13). In another study, the stigma of depression among medical, technical, and art students, and their attitudes toward seeking help were examined. It showed that the most common way students became familiar with mental health conditions was by watching videos and films related to mental illness (14). Amini et al. (15) examined medical students' views on psychiatry and its selection as their future field. In this study, about half of those who expressed interest in psychiatry had relatives or close friends with a psychiatric disorder (15). In another study, the effect of increasing medical students' exposure to people with mental illness was examined, which showed that the increased exposure did not improve students' negative attitudes toward mental illness and psychiatric conditions (16).

Most studies emphasize the importance of psychiatric education to reduce stigma, especially among medical students. However, the findings from previous studies are scattered and have not yet been presented in a coherent program or package to reduce stigma. For this reason, insufficient information is available on the implementation of interventions and their impact over a long period of time. Since there are no established educational program to reduce stigma through medical educational system in Iran, we aimed to provide a practical and effective training package to reduce stigma of people with mental illness in medical students using an expert panel with Delphi method, based on a scoping review.

## Materials and Methods

### Step 1: Search Strategy, Data Extraction and Quality Assessment

We searched the available international databases including PubMed, Google Scholar, Scopus, PsycINFO, Tripdatabase, Web of Science, Cochrane Database of Systematic Reviews as well as Persian databases including Iranmedex, SID, Irandoc, and Magiran. We did not limit our searches to a specific time period. The languages of the searched sources were English and Persian. Persian equivalent terms for stigma, attitude, mental illness, intervention, and program were used separately and combined in the Iranian bibliographic database. In the International Bibliographic Database, the keywords of stigma and related words, mental illness and related words such as intervention, reduction, improvement, or similar words were used in combination. We combined the results of each search with the AND operator.

We performed the online search in February and March 2020 and retrieved a total of 3,070 articles and 200 theses. Two researchers reviewed each document independently and a third person reviewed the possible disagreements for a final decision. Initially 1,209 articles and 160 theses were excluded due to duplication. In the next stage, by reviewing the titles, 1,692 articles and 36 theses were excluded due to lack of relevance with the study's objectives. Then, we reviewed the abstracts and excluded 148 articles and three theses. Finally, 21 article full texts and one thesis were thoroughly reviewed for inclusion criteria.

**Figure 1:**
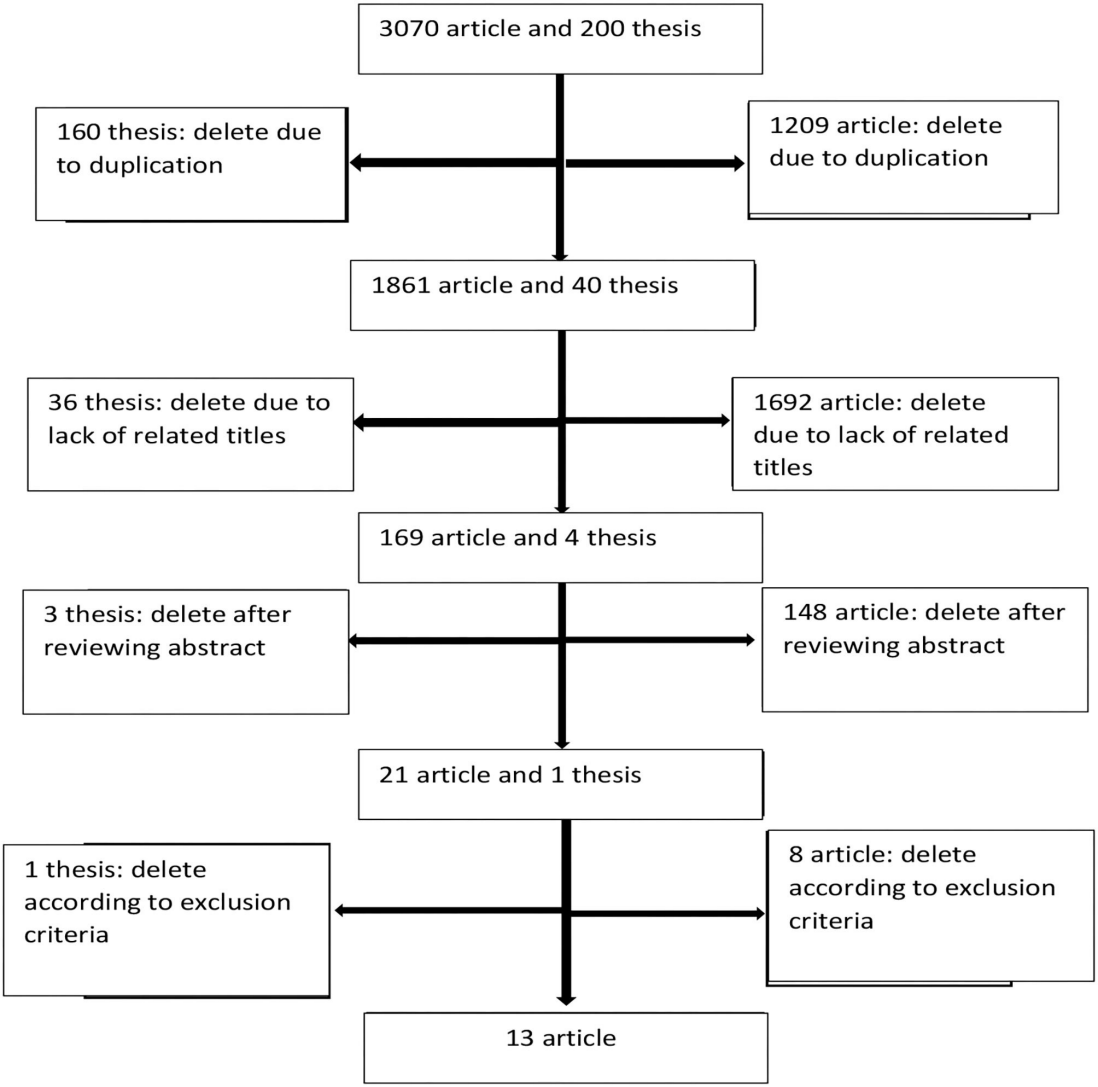
The methodology for finding and narrowing down relevant articles and theses.

Out of a total of 21 articles and one thesis, 13 articles were selected according to our inclusion criteria (Figure 1). We included studies that assessed the efficacy of interventions to reduce stigma of mental illness in health system staffs (physicians, nurses, psychologists, social workers and health system students), with a randomized controlled trial method.

We extracted the type of stigma reduction interventions, the sample sizes of the control and intervention groups, the mean stigma scores in each group before and after the intervention, the standard deviations and the durations of the interventions. Then, the effect size of each intervention was calculated using Cohen's formula. The data obtained from each intervention are provided in Table 1. Finally, out of 13 selected articles, 19 stigma reduction interventions were obtained to enter the expert panel using Delphi method.

**Table 1:** Anti-stigma interventions and their effect in previous studies.

**Title of study**	**Intervention**	**Follow up and the duration of the intervention**	**The effect size of the intervention**	
			**Control group**	**Intervention group**
Randomized study of different anti-stigma media Finkelstein et al. (17)	1. Reading articles related to stigma 2. Holding training sessions through the media	1. Immediately after the intervention 2. Six months after the intervention	*N* = 48 CAMI (Authoritarianism) → after intervention: ES^*a*^: 0 Six months after intervention: ES: 0.25 CAMI (Benevolence) → After intervention: ES: 0 Six months after intervention: ES: 0.09 CAMI (Social restrictiveness) → After intervention: ES: 0 Six months after intervention: ES: 0.04	Reading group (*n* = 76), Program group (*n* = 69) Reading group: CAMI^*b*^ (Authoritarianism) → after intervention: ES: 0.65 Six months after intervention: ES: 0.36 CAMI (Benevolence) → After intervention: ES: 0.37 Six months after intervention: ES: 0.09 CAMI (Social restrictiveness) → after intervention: ES: 0.72 Six months after intervention: ES: 0.16 Program group: CAMI (Authoritarianism) → after intervention: ES: 1.42 Six months after intervention: ES: 0.84 CAMI (Benevolence) → after intervention: ES: 0.8 Six months after intervention: ES: 0.41 CAMI (Social restrictiveness) → after intervention: ES: 1.39 Six months after intervention: ES: 0.58
Putting the person back into psychopathology: an intervention to reduce mental illness stigma in the classroom Mann et al. (18)	Holding classes and training based on the humanism of the disease along with presenting the life story of a psychiatric patient	No follow up	Control group: *n* = 26 ES: 0.0	Experimental group: *n* = 27 ES: 2.83
Changing Stigma Through a Consumer-Based Stigma Reduction Program Michaels et al. (19)	Holding training workshops, training with contact with people	No follow up	Control group: *n* = 65 ES: 0.1	Interventional group: *n* = 65 ES: 0.30
Comparing the Effect of Contact-based Education with Acceptance and Commitment Training on Destigmatization Toward Psychiatric Disorders in Nursing Students Vaghee et al. (20)	1. Training along with contact with psychiatric people who have improved 2. ACT	1. After the intervention 2. One month later	Control group: *n* = 36 After intervention: ES: 0.37 One month after intervention: ES: 0.63	Contact-based education: *n* = 37, ACT^*c*^ group: *n* = 38 Contact-Based Education Group: After intervention: ES: 1.08 One month after intervention: ES: 1.85 ACT Group After intervention: ES: 0.89 One month after intervention: ES: 1.28
Comparing the effects of live and video-taped Theatrical performance in decreasing Stigmatization of people with serious mental illness Faigin et al. (21)	1. Watching theater with the subject of stigma related to the person with a mental health condition 2. Watching movies	1. After the intervention 2. One month later	Control group: *n* = 123 CAMI (Authoritarianism) → After intervention: ES: 0.15 One month after intervention: ES: 0.20 CAMI (Social Restrictiveness) → After intervention: ES: 0.16 One month after intervention: ES: 0.24 CAMI (Social Restrictiveness) → After intervention: ES: 0.07 One month after intervention: ES: 0.02	Live group (theater): *n* = 81, video group: *n* = 99 Live group (Theater): CAMI (Authoritarianism) → After intervention: ES: 0.31 One month after intervention: ES: 0.14 CAMI (Social Restrictiveness) → After intervention: ES: 0.26 One month after intervention: ES: 0.13 CAMI (Benevolence) → After intervention: ES: 0.3 One month after intervention: ES: 0.09 Video group: CAMI(Authoritarianism) → After intervention: ES: 0.04 one month after intervention: ES: 0.10 CAMI (Social Restrictiveness) → After intervention: ES: 0.02 one month after intervention: ES: 0.17 CAMI (Benevolence): After intervention: ES: 0.1 one month after intervention: ES: 0.07
Filmed v. live social contact interventions to reduce stigma Clement et al. (22)	1. Watch a movie of health care providers talking about their experience with a patient with a psychiatric disorder; Watch a movie of people telling their life story and their experience of stigma 2. Watch a lecture of a patient and therapist about their experience of mental health and stigma	1. After the intervention 2. Four months later	Lecture (control) group (*n* = 124) After intervention: ES: 0.26 Four month after intervention: ES: 0.19	DVD group (*n* = 117), Live group (*n* = 119) DVD group: After intervention: ES: 0.79 Four months after intervention: ES: 0.54 Live group: After intervention: ES: 0.65 Four months after intervention: ES: 0.22
Impact of Skill-Based Approaches in Reducing Stigma in Primary Care Physicians Beaulieu et al. (23)	Proficiency in diagnosing and treating depression and anxiety and self-confidence in patient management	No follow up	Control group (*n* = 34) ES: 0.10	Intervention group (*n* = 39) ES: 0.32
A mental health training program for community health workers in India: impact on recognition of mental disorders, stigmatizing attitudes and confidence Hofmann-Broussard et al. (24)	Holding a workshop (introducing mental health and its disorders with questions and answers, promoting mental health in the community, improving communication, direct contact with people, direct contact with the improved patient and hearing their life story)	No follow up	Control (*n* = 22) Stigma score–psychosis vignette: ES: 0.25 Stigma score–depression vignette: ES:0.48	Intervention (*n* = 34) Stigma score–psychosis vignette: ES: 0.84 Stigma score–depression vignette: ES: 0.58
Effects on Knowledge and Attitudes of Using Stages of Change to Train General Practitioners on Management of Depression Shirazi et al. (25)	Holding a workshop (teaching the diagnosis and treatment of people with depression), showing movies and discussing it, forming open groups, presenting and introducing the patient, playing the role of the patient-therapist	No follow up	ES: 1.44 (Size of the intervention effect on physicians' awareness)	ES: 2.09 (Size of the intervention effect on physicians' awareness)
Reducing the Mental Health–Related Stigma of Social Work Students Rubio-Valera et al. (26)	Educating and sensitizing students to psychiatric problems along with holding a workshop by a patient with a psychiatric disorder who has had ten training sessions about empowerment and learning communication skills with a social worker	1. Fifteen days later 2. Three months later	Intervention (*n* = 79) CAMI (Authoritarianism) → After 15 days: ES: 0.16 After 3 months: ES: 0.30 CAMI (Benevolence) → After 15 days: ES: 0.33 After 3 months: ES: 0.30 CAMI (SCMHC) → After 15 days: ES: 0.33 After 3 months: ES: 0.42 Personal stigma: After 15 days: ES: 0.22 After 3 months: ES: 0.40 Perceived stigma: After 15 days: ES: 0.08 After 3 months: ES: 0.12	Intervention (*n* = 87) CAMI (Authoritarianism) → After 15 days: ES: 0.46 After 3 months: ES: 0.45 CAMI (Benevolence) → After 15 days: ES: 0.47 After 3 months: ES: 0.26 CAMI (SCMHC) → After 15 days: ES: 0.63 After 3 months: ES: 0.39 Personal stigma: After 15 days: ES: 0.50 After 3 months: ES: 0.24 Perceived stigma: After 15 days: ES: 0.06 After 3 months: ES: 0.11
Reducing the stigma of mental illness in undergraduate medical education Papish et al. (27)	Contact with a patient with a psychiatric disorder	1. After the intervention 2. The end of the training course 3. Three months later	Control group (*n* = 56) After intervention: ES: 0.12 End of training course: ES: 0.45 After 3 months: ES: 0.45	Intervention group (*n* = 55) After intervention: ES: 0.05 End of training course: ES: 0.61 After 3 months: ES: 0.48
Anti-stigma films and medical students' attitudes toward mental illness and psychiatry Kebry et al. (28)	Showing Anti stigma movies	1. After intervention 2. Eight weeks later	Control group (*n* = 23) After intervention: ES: 0.02 Eight weeks later: ES: 0.22	Intervention group (*n* = 23) After intervention: ES: 0.38 Eight weeks later: ES: 0.08
The effect of an anti-stigma program on stigma components on people with a mental health condition among nursing students Asayesh et al. (29)	Anti-stigma program (training program, teaching communication skills to people, group meeting with emphasis on identifying negative thoughts and beliefs and applying the principles of cognitive therapy, group therapy with stigma-related topics for people in the psychiatric ward with the participation of students as a group member, direct contact with people, increasing students' skills in communicating with people	After the end of the training course	Control group (*n* = 23) ES: 0.02	Intervention group (*n* = 20) ES: 1.76

*^1^Effect Size*.

*^2^Community Attitude Mental Illness Score*.

*^3^Acceptance and commitment therapy*.

### Step 2: Delphi Expert Panel

The collected data were entered into the Delphi method to select interventions to design an educational package for improving attitudes toward people with mental illnesses amongst Iranian medical students. In this way, sixteen experts, faculty member with a substantial research and practical history on the topic, from different related fields such as psychology, psychiatry and social medicine were invited to participate in the Delphi process. The selection of experts was based on one of the following criteria: 1. Experts with at least 15 years of work in the field. 2. Who have research and practical experience on the stigma toward patients with mental illness topic. 3. These individuals were invited to participate and cooperate in selecting the best educational interventions to reduce stigma. In this way, the data collected from the review of previous studies (Table 1) and a list of interventions were sent via email to the participating experts. They were asked to score each intervention, based on submitted evidence, feasibility criteria, degree of necessity, degree of attractiveness for students, and the possible impact on the Likert Scale from one (strongly disagree) to four (Strongly agree. At the end of the list of interventions for scoring, two open-ended questions were asked from experts about the ability to combine interventions and indicate the most appropriate combination of interventions to be included in the educational package. Out of 16 experts, 14 experts sent back their answers. Experts' responses were summarized both quantitatively (Table 2) and qualitatively. In the quantitative method, the total scores given in the subscales defined for each intervention (feasibility, degree of necessity, level of attractiveness for students and potential impact) were averaged. In the qualitative method, the opinions for and against each intervention method and the experts' answers to the open questions were collected and organized. In the next step, the authors discussed the quantitative and qualitative responses to select interventions for the development of the educational package. As a result, the interventions with a low score (average score ≤3) and interventions that were argued to be less adaptable to Iran's cultural and social conditions were excluded. Finally, out of 19 interventions, six were prioritized. The six selected interventions employed one or more of the following four approaches; direct education, contact with people with mental disorders, video screening, and group discussions.

**Table 2:** Quantitative average of data in the first stage of Delphi.

**Intervention no**.	**Intervention type**	**Feasibility**	**Necessity**	**Attractiveness for the student**	**Possible impact in case of implementing**
1	Holding training sessions through the media	3.60	3.49	2.74	2.78
2	Reading articles related to stigma	3.22	2.74	1.92	2.17
3	Holding a training class based on the humanity of psychiatric people	3.20	3.35	2.78	2.92
4	Holding a workshop	3.28	3.28	3	2.92
5	Education with contact with people	3.20	3.63	3.42	2.99
6	Educate and sensitize students about psychiatric problems	3.35	3.35	2.64	2.49
7	Playing the role of patient-therapist	3.01	3.09	3.42	2.84
8	ACI	2.35	2.49	2.67	2.74
9	Being proficient in diagnosing and treating depression and anxiety and self-confidence in patient management	3.42	3.28	3	2.78
10	Showing movies about social stigma	3.56	3.17	3.56	2.92
11	Presenting the life story of a psychiatric patient by students	3.06	2.92	3.07	2.70
12	Direct face-to-face contact with a patient with a psychiatric disorder	3.77	3.49	3.09	2.92
13	Watch a video of health care providers (with the content of presenting their experience with a person with a mental health condition) and watch a video of a patient with a psychiatric disorder (with the content of presenting their experience of stigma and their life story)	3.49	3.21	3.28	2.78
14	Watch live patient and therapist lectures on expressing their experience about mental health and stigma	3.34	3.07	3.13	2.92
15	Identify students' negative thoughts about the mentally ill and reform misconceptions	2.92	3.17	3.17	2.74
16	Formation of open groups and discussion about Stigma	3.42	3.31	3.20	2.99
17	Group therapy with stigma-related topics for people with a psychiatric disorder with the participation of students as active members of the group	2.31	2.88	2.85	3.07
18	Workshop by a patient with a psychiatric disorder trained in empowerment and learning skills.	2.45	2.53	2.85	2.63
19	Theatrical performances with the theme of stigma related to the mental illness	2.49	2.28	2.99	2.63

In the second stage of the Delphi process, the 14 experts who collaborated in the first stage of Delphi, were asked to comment on implementing the six interventions selected for the educational package and explain their reasoning for agreement or disagreement. Out of the 14 experts, 11 experts submitted their answers at this stage. The responses were summarized and the final findings were reviewed and discussed by the authors in this study.

## Results

After reviewing the 13 selected articles, the type of stigma reduction interventions, the impact of the interventions, the follow-up period after the interventions, and the durations of the impact of the interventions were extracted from the available articles. Most studies used more than one intervention or combination of several interventions to reduce stigma on the target group. The findings of previous studies are presented in the Table 1 below.

The findings obtained in this study are discussed below:

### Qualitative and Qantitative Summary of Data Obtained From the First Stage of Delphi

This stage is the result of the findings obtained from the first Delphi stage. The consensus among all the participants in the first stage of Delphi was that all of these interventions could be combined, people and if implemented properly, can be effective in reducing stigma among Iranian medical students. One participant stated that this work should not be limited to one course or 1 month of training and should last for 3–5 years during the students' theoretical, internship, and clinical training courses to achieving higher impact and quality. Some participants emphasized implementing approaches that include the three areas of knowledge, cognition, and behavior. One participant believed that stigma is a kind of phobia that can be reduced through repeated exposure. Of the 19 interventions obtained, most participants suggested interventions that include theoretical training and increased interaction and social contact.

### Data From the Group Discussion of Authors After the First Stage of Delphi

The researchers of this study discussed the data obtained from the first stage of Delphi. Based on the findings, six stigma reduction interventions were selected for the proposed education package, consisting of holding a workshop, education with contact with people, training in diagnosis and treatment of depression and anxiety and self-confidence for patient management, showing movies about social stigma, direct face-to-face contact with a people with a psychiatric disorder, and the formation of open groups and discussions about stigma.

### Data From the Second Stage of Delphi

The experts re-evaluated the six selected stigma reduction interventions in the second stage of Delphi. At this stage, participants explained their agreement or disagreement. In the second Delphi stage, participants commented on each of the interventions, which is given below:

#### Intervention of Holding a Workshop

Three experts disagreed with its implementation in the stigma reduction educational package. The first participant who opposed this method believed that it is an efficient, practical, attractive, and innovative method, but most professors are unfamiliar with its design and implementation. It is also very time-consuming and costly, and with the financial problems of hospitals and the education system in Iran, there is no priority for holding a workshop. Another expert explained that only workshops with motivational goals could lead to change, which is not a conventional standard for workshops in Iran. A third person opposed to this method believed that a workshop is practical as a part of the de-stigmatization method and that it is not enough to change attitudes and behavior on its own. Eight experts agreed with the implementation of this intervention in the training package. However, to increase the effectiveness, most of them emphasized two-person active interaction in the workshop, using educational posters in the sessions, and holding the workshop by professional instructors in this field.

#### The Training Intervention Along With Contact With People

only one of the experts disagreed with this intervention who believed that despite the necessity of this intervention, due to its implementation in the current educational curriculum for students, it will not introduce a change in the existing process in the new educational package. Other experts agreed with this intervention. One of the participants suggested this intervention as the priority in the educational package. Most of the experts believed that this intervention should be implemented in outpatient centers, counseling, rehabilitation, private psychiatric clinics, and centers outside the hospital where most people are Non-emergency, Non-psychotic, and have psychosomatic and Non-chronic illnesses, in order to increase the effectiveness of this intervention for students and minimize the risk for people.

#### Diagnosing and Treating Depression, Anxiety, and Self-Confidence Training for Patient Management

Two experts disagreed with implementing this intervention in the training package. One of the experts believed that implementing this intervention, despite its usefulness, is not feasible with the current facilities. The other expert stated that despite the usefulness of this method, due to doubts about its effect on stigma removal, it is not applicable. Other experts agreed with using this intervention in the educational package. Although many experts were unsure about the effectiveness of this intervention in reducing stigma, they nonetheless believed that this method could improve students' learning by increasing student's empathy skills with people and help provide a comprehensive understanding of people. One of the experts mentioned the necessity of implementing this intervention and emphasized holding regular student communication sessions with the patient in the presence of an educational supervisor to identify the student's problems and mistakes in managing the patient's diagnosis and treatment and give them relevant training.

#### Intervention of Showing Movies About Stigma

Two experts believed that showing movies have no lasting effect on reducing stigma and disagreed with its effectiveness. One of the participants had no opinions on this intervention due to the lack of information about stigma reduction movies that are culturally appropriate and relevant in Iran. Other participants agreed with the inclusion of this intervention in the training package as an auxiliary method due to its educational appeal to students and to help them learn better.

#### The Direct Contact and Face-To-Face Intervention With People Who Affected Psychiatric Disorders

Only one of the experts disagreed with its implementation, who believed that contact with people who affected psychiatric disorders already exists in ongoing courses of students and will not have much effect. Other experts agreed with its implementation in the educational package, and one expert suggested it as the priority of interventions in the educational package. Many experts believed that this intervention would be effective if it were implemented after students' course work is completed and in centers where most of the people are Non-emergency, Non-psychotic, and Non-chronic. Direct contact with people will also lead to a deeper understanding by students of people' circumstances. One expert suggested that people be selected from different social and cultural classes.

#### Open Group Intervention and Discussion About Stigma

All experts agreed to implement this measure. Some of them suggested it as a necessary component and the most appropriate choice for the educational package. Most participants believed that this method would be very effective if directed well. Suggestions were made to better implement this intervention, including training in group discussion, group participation, group counseling, and problem-solving training. Its implementation should be conducted under the direction of trained educators and facilitators skilled in interactive work. One of the experts suggested that if participation in these group discussions were optional, its impact would increase.

### Data From the Group Discussion of Rsearchers After the Second Stage of Delphi

Findings obtained from the second stage of Delphi were again shared among the authors in this study for the final selection of appropriate interventions to develop a stigma reduction package. After examining the ideas of experts, researchers concluded that the training interventions for the proposed educational package should be presented in an interactive and multi-stage combination. The combination of interventions can have a strengthening effect on the effect size and increase the level of involvement of people in education. The authors omitted the workshop intervention from the training package due to the difficulties in the educational system for holding workshops and the disagreements of some experts about it. Since diagnosing and treating depression, anxiety, and self-confidence for patient management is better taught to students by watching a diagnostic and therapeutic interview by a professor or assistant, it is unnecessary to place it as a separate intervention in the stigma reduction educational package. Instead, it should be implemented as an essential educational component and other interventions in the students' education to improve the mental health system and the quality of education. Finally, effective methods for reducing stigma and generalities of the educational package were presented with four interventions including, film screening, education through contact with people with psychiatric disorders, contact with people with psychiatric disorders, and group discussion on stigma definition and personal experiences were designed as an interactive and multi-stage combination as follows:

The First Stage:
Screening movies related to stigma, holding group discussion on defining stigma, and participants' personal experiences.
The appropriate time for implementing the first stage of the educational package is in the first week of the students' training course for a minimum of 2 h.
The Second Stage:
Training by psychiatry faculty members along with contact with people: Visiting people by faculty members in outpatient and Non-emergency inpatient centers in the presence of students and at the same time giving the necessary training about the illness and how to communicate with a patient with a psychiatric disorder.
This stage is performed daily during a 1-month training course for students. In the absence of faculty members, this training will be continued by a psychiatric trainee.
The Third Stage:
Contact with people with psychiatric disorders: During this training course, students can independently obtain clinical and community history from two people with psychiatric disorders who are outpatient or hospitalized in psychosomatic and Non-emergency psychiatric wards. Also, for contact with Non-emergency people with psychiatric disorders, daily centers are recommended. Contact with patients refers to students' active participation in patients' classes at day centers, visiting patients' handicrafts shows, and talking directly with patients to hear their life stories.
The Fourth Stage:
Group discussion on defining stigma and personal experiences: It‘s preferred to be led by a faculty member as well as a trained medical student as co-facilitator. Atmosphere of the group must be easy and Non-judgmental and almost all time should be divided for sharing experience of every student and intervention of the leaders should be remained at minimum level. Suggested duration of the group discussion is 90–120 min.

## Discussion

The issue reviewed in the present study is the importance of implementing interventions whose research evidence has shown their effectiveness and usefulness. In addition, these interventions needed to be adaptable to Iran's specific cultural, social, and economic conditions and implementable in the education system. According to previous studies, film screening is an effective intervention in reducing stigma, but most of the studies emphasized its effect on reducing stigma in a short period, and there was no evidence of its long-term effect (18, 21, 25, 28, 30, 31). The researchers in this study, despite knowing of the short-term effect of a film screening in reducing stigma, recommended it as a means to attract students' attention and increase their motivation to participate in the intervention program. The advantages of this intervention method are educational attractiveness, low cost and participants' reflections on it in discussions and subsequent sessions about that movies, and finally, its feasibility of implementing this measure in the educational system.

Regarding the group discussion intervention, all the experts agreed with this measure in the stigma reduction educational package, and some suggested it as the priority in the educational package. In the study of Shirazi et al. (25), the formation of open groups and group discussions in combination with other interventions created a significant improvement in the knowledge and awareness of physicians. As a result, it was recommended to change the educational method to increase awareness and change physicians' behavior to improve communication skills with people with psychiatric disorders. In the study of Asayesh et al. (29), open group discussions about stigma for people in the psychiatric ward were accompanied by students' participation as active members of the group and, combined with other interventions, reduced stigmatization of people among students. In a review study by Heim et al. (32) on open group discussion intervention, one study demonstrated a positive effect on students' attitudes toward psychiatry but did not change their attitudes toward people with psychiatric disorders. In another study, open group discussion combined with other interventions was effective on students' attitudes toward people with psychiatric disorders. According to previous findings, group discussion in combination with other interventions can positively affect changing attitudes and reduce stigma toward people with psychiatric disorders. The researchers of this study also believed in its effectiveness in combination with other educational interventions to reduce stigma, with content about discussions of stigma issues and personal experiences of stigma. One of the advantages of this method is its feasibility in the departments and colleges, and availability. It will also help students pose questions and increase their awareness.

Previous studies have shown that the combination of education and contact with people has effectively reduced negative attitudes and increased people's awareness but has been less effective in changing people's behavior with these people. It is also short-term and less effective over a long period of time (16, 19, 20, 26, 33, 34). The type of contact with people in these studies was through simultaneous training during patient visits, training in the presence of a patient with a psychiatric disorder who has improved, employment in psychiatric wards, providing a theory course at the time of a psychiatric internship, face-to-face interview with the patient in the presence of the instructor. The researchers of this study placed this intervention in the second stage of the proposed educational package and suggested students' contact with patients in this stage through faculty members' visits with patients in the presence of students along with direct education on mental disorder and the impact of biological and environmental causes on them. Furthermore, diagnosis and treatment skills and self-confidence are taught to manage people, which is necessary to promote mental health and increase the quality of treatment of people with psychiatric disorders. There is evidence of the impact of contact with people with mental disorders in previous studies. Interventions based on social interaction with people were the most effective way to improve attitudes and increase interest in communicating with people with psychiatric disorders (20, 31, 34, 35). According to a review by Thornicroft et al. (36), social interaction-based interventions usually improve attitudes in the short term. In a review study by Heim et al. (32), communicating with people directly or visually was associated with improved students' attitudes toward people. In a review study by Mehta et al. (37), in a short period of time, communicating with psychiatric people was more effective in reducing stigma than other intervention methods. More contact with people with severe, chronic, and refractory disorder may have the opposite effect. In the study of Amini et al. (16), contact with people with psychiatric disorders did not affect students' negative attitudes, contrary to the data from Western studies, which may be due to students' contact with people with severe mental disorders in psychiatric wards.

The researchers of this study recommended face-to-face contact with people with psychiatric disorders in the third stage of the educational package after the combined training and contact intervention (in the second stage of the educational package). Contact with people at this stage was suggested by attending day centers, Non-emergency wards, or outpatient centers.

### Limitations

1. One of the limitations of this research was that in searching databases and reviewing journals manually, there might be articles under publication but have not yet been registered in the database or hidden from the researcher and entered into the study.
2. Due to the limited workforce and the financial crisis of the Iranian educational system, it was not possible to include some effective interventions in studies abroad, including a workshop and holding a conference to reduce stigma.
3. In many follow-up studies, the effect of the intervention over a long period of time has not been reported.
4. The effect of interventions in most studies has been measured in combination with other interventions, and there is not enough information about the effect of each intervention separately.
5. Lack of cooperation of some experts in the second stage of Delphi.

### Research and Practical Recommendations

1. To determine the appropriate content for the implementation of this educational package, including selecting the appropriate videos available to reduce stigma by experts; preparing appropriate videos to reduce stigma; including providing a video of a professor's psychiatric interview with a patient with his her informed consent; preparing a video about a personal experience of being stigmatized by a person who is recovering from a psychiatric disorder story with his/ her informed consent; inviting a celebrity figure in the field of science or art who suffers from a psychiatric illness to present a lecture on their experience and its management; provide opportunities for students to visit the activities of people with psychiatric.
2. Implementing some stigma reduction interventions, not limited to the medical students' psychiatric training course.
3. Evaluation of the effectiveness of this package in the target group.
4. Designing suitable packages for students and people working in other medical professions such as occupational therapy and nursing.
5. Coordinated efforts to fund research and support investment in stigma reduction interventions.

## Conclusions

Because among Iranian mental health professionals and planners, there is no coherent and targeted program to reduce stigma and subsequently eliminate the burden of the illness, in the present study, we present interventional methods to reduce stigma in the form of four intervention methods, as a combination, interactive which include: 1. Film screening, group discussion on it, 2. Education with contact with people with psychiatric disorders, 3. Contact with people with psychiatric disorders, 4. Group discussion on defining stigma and personal experiences.

## Data Availability Statement

The raw data supporting the conclusions of this article will be made available by the authors, without undue reservation.

## Ethics Statement

This article has been extracted from the approved plan with ethics code IR.IUMS.REC.1397.997 on 28/10/2018 by Institute Review Board of Iran University of Medical Sciences. Written informed consent for participation was not required for this study in accordance with the national legislation and the institutional requirements.

## Author Contributions

SS, MS, RS, and MR: conceptualization and design. FR, MS, and MR: data collection and initial draft preparation. All authors editing and review. All authors contributed to the article and approved the submitted version.

## Conflict of Interest

The authors declare that the research was conducted in the absence of any commercial or financial relationships that could be construed as a potential conflict of interest.

## Publisher's Note

All claims expressed in this article are solely those of the authors and do not necessarily represent those of their affiliated organizations, or those of the publisher, the editors and the reviewers. Any product that may be evaluated in this article, or claim that may be made by its manufacturer, is not guaranteed or endorsed by the publisher.
